# Effect of panretinal photocoagulation versus intravitreal bevacizumab injection on optic disc microcirculation in patients with diabetic retinopathy

**DOI:** 10.1186/s40942-024-00621-w

**Published:** 2024-12-18

**Authors:** Hassan Khojasteh, Mohammad Ahadi Fard Moghadam, Masoud Rahimi, Arash Mirzaei, Fariba Ghassemi, Alireza Takzare, Hooshang Faghihi, Elias Khalili Pour, Hamid Riazi-Esfahani

**Affiliations:** 1 Retina Service, Farabi Eye Hospital, Tehran University of Medical Sciences, South Kargar Street, Qazvin Square, Tehran, Iran; 2 Anaesthesiology Department, Farabi Hospital, Tehran University of Medical Sciences, Tehran, Iran

**Keywords:** Diabetic retinopathy, Optical coherence tomography angiography (OCTA), Vascular density, Panretinal photocoagulation (PRP), Anti-vascular endothelial growth factor (anti-VEGF), Retinal nerve fiber layer (RNFL) thickness

## Abstract

**Background:**

This retrospective study aimed to compare optic disc vasculature changes in 1 and 3 months after treatment with either panretinal photocoagulation (PRP) or Intravitreal bevacizumab (IVB) in patients with diabetic retinopathy.

**Methods:**

A total of 50 eyes of 29 diabetic patients without severe complications were included in this comparative case series. Of these, twenty-eight eyes (15 patients) were assigned to the PRP group, while twenty-two eyes (14 patients) were treated with the biosimilar (IVB) (Stivant^®^ CinnaGen Co., Iran). Optical tomography angiography (OCTA) was performed to measure optic disc vascular density (VD) as well as retinal nerve fiber layer (RNFL) thickness.

**Results:**

The mean age of the enrolled patients was 62.1 ± 8.3 years (40 to 78 years). During follow-up, whole disc VD, inside disc VD and peripapillary VD decreased significantly in the PRP group at month 1 (*p* = 0.032, *p* = 0.32, and *p* = 0.016, respectively) and month 3 (*p* = 0.004, *p* = 0.001 and *p* = 0.011, respectively). There was an insignificant and slight increase for these parameters in the IVB group. (*p* > 0.05 for all) A comparison of two treatment arms based on mean whole disc VD, inside disc VD, and peripapillary VD changes revealed a significant difference at month 1 (*p* = 0.009, *p* = 0.019, and *p* = 0.002, respectively) and month 3 (*p* = 0.002, *p* = 0.015, and *p* = 0.009, respectively). Peripapillary RNFL thickness increased in the PRP group at month 1 (*p* = 0.002) and then decreased at month 3 (*p* = 0.001). During three months of follow-up, the peripapillary RNFL thickness decreased significantly in the IVB group (*p* = 0.001). Peripapillary RNFL thickness changes were significantly different between treatment groups at month 1 and month 3. (*p* = 0.001 for both) The RNFL changes during the study did not significantly correlate with peripapillary VD changes in each group (*p* = 0.231 and *p* = 372, for PRP and IVB group, respectively).

**Conclusion:**

This study demonstrated that IVB and PRP treatments produced distinct short-term microvascular changes in the optic nerve of diabetic retinopathy patients. PRP treatment significantly reduced vascular density in the optic disc and peripapillary region over 3 months, with an initial increase in RNFL thickness followed by a decrease by month 3. In contrast, IVB treatment led to a slight increase in optic disc vascular density while significantly reducing RNFL thickness. No significant correlation was found between changes in RNFL thickness and peripapillary vascular density within either treatment group.

## Background

Diabetic retinopathy (DR) is one of the complications of diabetes mellitus (DM), which can lead to blindness due to secondary side effects such as diabetic maculopathy and proliferative diabetic retinopathy (PDR) [1]. The main established treatments of proliferative diabetic retinopathy (PDR) are pan-retinal photocoagulation (PRP) and anti-vascular endothelial growth factor (VEGF) injections [2].

For decades, PRP has been the mainstay of treatment for PDR, which has been clinically proven to be effective in reducing the risk of vision loss for decades [3]. However, the laser is a destructive process and can lead to functional and anatomical impairments, such as worsening of macular edema, diminished visual acuity, visual field loss, and of night vision disturbance [4]. Anti-vascular endothelial growth factor (Anti-VEGF) is non-inferior to PRP in the treatment of PDR and also minimizes the risk of side effects associated with PRP [5]. Less visual field loss and less occurrence/worsening of diabetic macular edema (DME) were benefits of anti-VEGF compared to PRP that nowadays clinicians’ and patients’ preferences are the determinants of whether to treat severe non - proliferative diabetic retinopathy (NPDR) or PDR with anti-VEGF or PRP.

The effect of PRP on the optic disc is not fully understood, different studies demonstrated mixed results showing an increase, decrease, and no change in RNFL thickness after treatment [6–9]. The reduction of the optic disc blood flow has been shown during the 12 weeks after completion of PRP using a laser speckle flowgraph [10]. There is no conclusive study on the effect of Anti-VEGFs on optic disc microcirculation.

Optical coherence tomography angiography (OCTA) is a non-invasive and novel imaging modality of the retinal and optic disc vasculature that provides images of the different vascular layers [11]. The quantitative parameters of OCTA, like vessel density (VD) can be used to evaluate vascular perfusion and DR progression [12, 13]. Prior research has documented alterations in vascular density (VD) within the macular and optic disc areas following administration of anti-vascular endothelial growth factor (anti-VEGF) or panretinal photocoagulation (PRP) therapy in patients with proliferative diabetic retinopathy (PDR). However, the findings have been inconsistent, and no investigation has yet compared the impact of these two treatment approaches on the normal microvasculature of the optic disc [14–19]. The retinal nerve fiber layer (RNFL) harbors a vascular network known as radial peripapillary capillaries (RPCs). The aforementioned structures exhibit a parallel and radial orientation and possess a length that exceeds the typical capillary lacking anastomotic connections [20, 21]. The density of RPC decreases in proportion to the severity of diabetic retinopathy, and this phenomenon has been shown to be associated with visual acuity, visual field, and retinal nerve fiber layer thickness [22–24]. The evaluation of RPC density has the potential to facilitate the investigation of alterations in the microvasculature, particularly in the context of DME. This condition can result in segmentation errors, misclassification of retinal layers, and subsequent inaccuracies in the measurement of macular vessel density (VD) when compared to eyes that are free of DME [25].

In this study, we aimed to evaluate and compare the very early (1 month) and early (3 months) course of optic disc microvasculature as well as retinal nerve fiber layer (RNFL) thickness changes in severe NPDR or early PDR treatment-naïve patients while initiating treatment with either PRP or anti-VEGF using OCTA.

## Methods

This study was a retrospective comparative case series, approved by the local institutional review board (IR.TUMS.FARABIH.REC.1400.038). The study protocol adhered to the tenets of the Declaration of Helsinki and all participants provided written informed consent before participating in the study. This study incorporated patients who were referred to Farabi Eye Hospital between September 2021 and December 2022, and who presented with severe non-proliferative diabetic retinopathy (NPDR) or early proliferative diabetic retinopathy (PDR) in the absence of neovascularization of the optic disc (NVD), and had a visual acuity of ≥ 0.2 logarithms of the minimum angle of resolution (LogMAR) (Snellen: ≤ 20/32).

Patients underwent a thorough, complete ophthalmic examination including previous clinical history, slit-lamp biomicroscopy, and indirect dilated ophthalmoscopy. A masked optometrist measured best-corrected visual acuity (BCVA) (using the Snellen chart), and the results were then converted to a logarithm of the minimum angle of resolution (LogMAR). Macular optical coherence tomography (OCT) using the RTVue XR 100 Avanti device (Optovue, Inc., Fremont, CA, USA) was performed at baseline, as well as at months 1 and 3.

The classification of DR was based on the assessment of color fundus images and ranges from ETDRS Level 53 (severe NPDR) to Level 60–65 (mild to moderate PDR) [26]. The classification of DR was confirmed with fluorescein angiography (FA). Individuals with HRC-PDR were not included in the study due to the potential for vitreous hemorrhage, which would lower the quality of the OCTA images, as well as the presence of NVD, which would interfere with optic disc vessel density calculation.

The exclusion criteria were: uncontrolled diabetes (defined as hemoglobin A1c (HbA1c) ≥ 8%), the presence of fibrovascular proliferation in the macular area and optic disc, the presence of NVD (either in funduscopy or FA) or vitreous hemorrhage, eyes with visual acuity less than 20/200 Snellen, eyes with a history of previous treatment with IVB or any kind of photocoagulation, eyes with a positive ophthalmologic surgical history such as cataract surgery or vitrectomy in the past 6 months, uveitis, any type of glaucoma, the presence of any optic disc anomaly, optic disc pallor or swelling, OCTA images with quality index < 4/10, and refractive error > + 3 and < − 3. Eyes with severe media opacity were also omitted due to their potential impact on image quality. Other criteria for exclusion included pregnancy, no consent, or poor compliance with the study.

### Optic disc imaging

To assess optic disc vascular changes as well as the RNFL thickness patients underwent OCTA with the RTVue XR 100 Avanti device (Optovue, Inc., Fremont, CA, USA) which uses a split-spectrum amplitude-decorrelation angiography (SSADA) algorithm to improve signal-to-noise ratio and quantify the flow. The different layers of the retina were segmented automatically after using the projection artifact removal (PAR) algorithm (integral module in Angio Analytics software (version 2017.1.0.151)). All automated segmentations of retinal layers in OCTA images were rechecked and segmentation errors were manually corrected by two retina experts (EKP and HRE).

The optic disc scan covered an area of 4.5 × 4.5 mm^2^ centered on the optic disc. The VD was defined as the proportion of the total area occupied by blood vessels. In the optic disc scan, the software automatically calculated the whole image VD (covering an area of 4.5 × 4.5 mm^2^), average VD within the optic nerve head (inside disc VD), and peripapillary VD (measured in a 750 μm-wide annulus extending outward from the optic disc boundary). The peripapillary VD was analyzed from the radial peripapillary capillary segment, extending from the internal limiting membrane (ILM) to the posterior boundary of the RNFL. The vessel density measurement was done after large vessel removal by built-in software.

The optic nerve head protocol was used to measure RNFL thickness. It consists of 12 radial line scans of 459 A-scans each and its measurements were taken along 13 circular B-scans with diameters of 1.3–4.9 mm manually positioned on the optic disc to create a peripapillary RNFL thickness map. The RNFL thickness measurement was generated from a 3.45-mm-diameter circle and is calculated as the difference in distance between the internal limiting membrane and the outer edge of the inner plexiform layer. The average RNFL thickness was used for analysis.

**Fig. 1:**
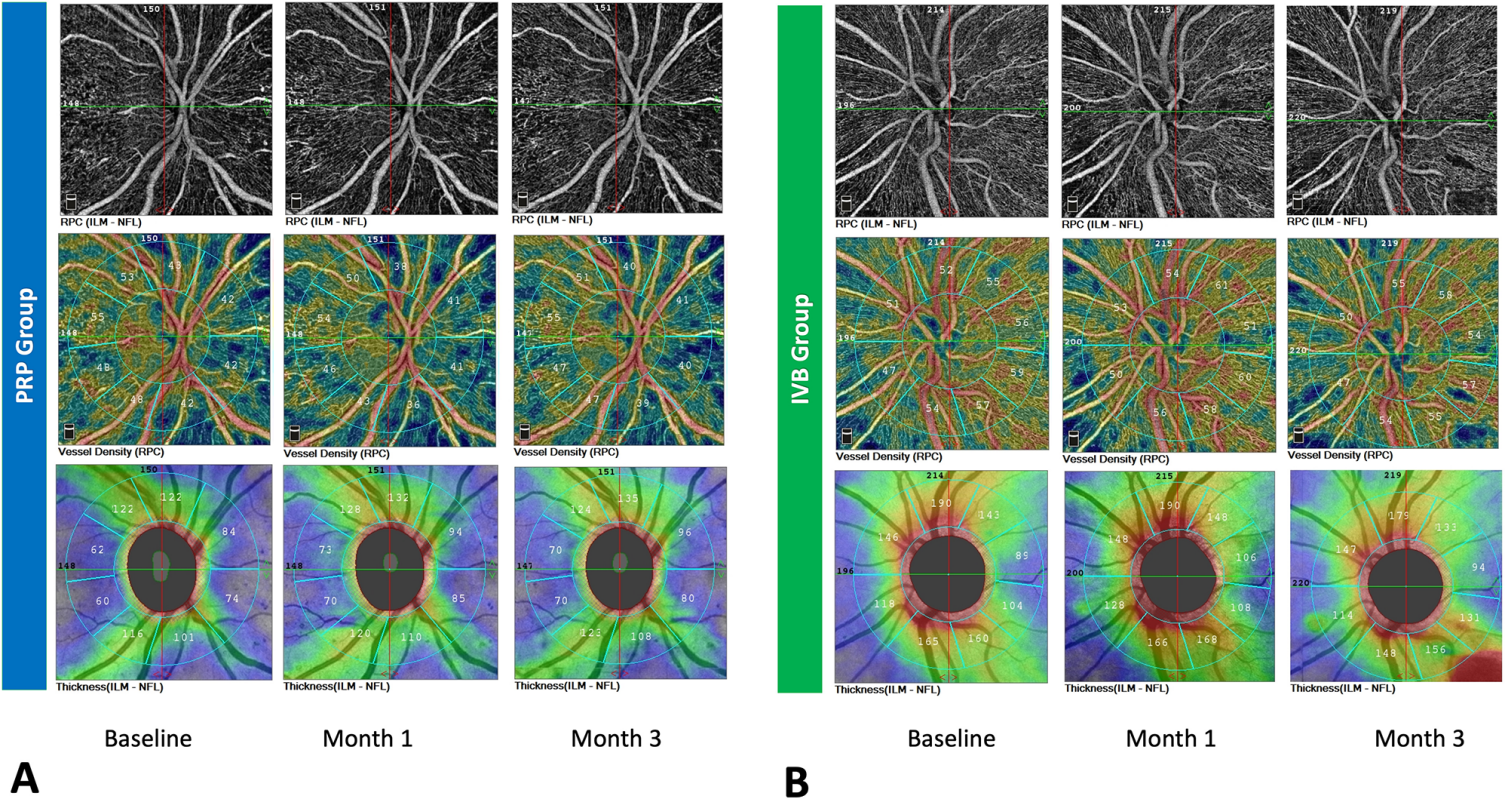
The Radial peripapillary capillary(RPC) density map (upper row), RPC density color-coded Map (middle row), and retinal nerve fiber layer (RNFL) color-coded thickness map (lower row) outlined at the baseline, 1 month and 3 months following pan-retinal photocoagulation (PRP) (**A**) and intravitreal bevacizumab (IVB) injections (**B**) for two different patients

Images were acquired at baseline, 1 and 3 months after completion of PRP, and during IVB treatments. (at baseline, 1 month after first injection and 1 month after third injection) The quality score $\:\ge\:$ 4/10 (according to the built-in RTVue software quality assessment) was accepted for image analysis. Poor quality images or images with artifacts such as shadows, defocus, motion, and decentration artifacts that prevented accurate measurement of the vascular density were excluded. (Fig. 1)

### Interventions

In the PRP group, eyes underwent PRP in two consecutive sessions (with an interval of 1 week) by an independent ophthalmologist according to guidelines published by the ETDRS research group; a topical anesthetic was used, and in each session, 1000 to 1200 Gy-white spots were made with an argon laser (532 nm) with a spot size of 500 mm, uniformly in all four quadrants [27]. The need for additional PRP was investigated at months 1 and 3 based on the presence of new neovascularization of the disc or elsewhere or new vitreous hemorrhage with visible fundus. In this group, intravitreal bevacizumab was injected after PRP when center-involved DME was present and vision, decreased to less than 20/25 after the month 3.

In the anti-VEGF group, eyes underwent three monthly intravitreal injections of bevacizumab biosimilar (IVB) (Stivant^®^ CinnaGen Co., Iran). This biosimilar drug has been investigated in previous studies [28, 29]. Intravitreal injections were performed in a sterile operating theater. Topical anesthetic drops were given first and then a lid speculum was inserted. After application of povidone-iodine 5% into the conjunctival sac for about 1-minute, intravitreal injection of 1.25 mg/ 0.05 ml bevacizumab was performed with a 29-gauge needle (1 ml tuberculin syringes; DispoVan) through the pars plana 4 mm and 3.5 mm posterior to the limbus in phakic and pseudophakic eyes, respectively. All patients were prescribed topical chloramphenicol 0.5% four times a day for five days after injection.

### Statistical analysis

All statistical analyses were performed by SPSS software (IBM Corp. Released 2020. IBM SPSS Statistics for Windows, Version 27.0. Armonk, NY: IBM Corp). Outcomes were reported as mean ± standard deviation (SD). To compare the demographic baseline variables between the two groups, we used t-test (quantitative normal data) and Chi-Square test. To compare the groups at different times considering probable inter-eye correlation, we used generalized estimating equation (GEE). Another GEE model was used to compare the parameters in different follow-ups within each group. To compensate for type I inflation due to multiple comparisons we used Sidak method. A p-value of less than 0.05 was considered statistically significant.

## Results

A total of 50 eyes (27 eyes with severe NPDR and 23 eyes with PDR) of 29 patients were included in the study. Of these, twenty-eight eyes (15 patients) were assigned to the PRP group, while twenty-two eyes (14 patients) were treated with the IVB. The demographic data of the patients are shown in Table 1. Fourteen patients (48.2%) were male. The mean age of the enrolled patients was 62.1 ± 8.3 years (40 to 78 years). There was no significant difference between the two treatment groups based on demographic data. Table 2 shows the baseline vascular densities as well as the baseline average RNFL thickness in the two groups. On the basis of measured baseline parameters, there was no significant difference between the two groups, except for inside-disc vascular density, which was substantially lower in the IVB group. (*p* = 0.017)

**Table 1:** Baseline demographic data of the patients in pan-retinal photocoagulation (PRP) and intravitreal bevacizumab (IVB) injection groups

Parameter		Total	Treatment		P
			PRP	IVB	
Age	Mean ± SD	62.1 ± 8.3	61.8 ± 8.9	62.5 ± 7.7	0.763†
	Median (range)	62 (40 to 78)	61.5 (48 to 78)	63 (40 to 72)	
Sex	Male	14 (48.2%)	9 (60.0%)	5 (35.7%)	0.166*
	Female	15 (51.7%)	6 (40.0%)	9 (64.2%)	
Laterality	OD	26 (52.0%)	15 (53.6%)	11 (50.0%)	0.802*
	OS	24 (48.0%)	13 (46.4%)	11 (50.0%)	

† Based on t-test

* Based on Chi-Square test

**Table 2:** Whole disc vascular density, inside disc vascular density, Peripapillary vascular density, and retinal nerve fiber layer (RNFL) thickness 1 month, and 3 months after pan-retinal photocoagulation (PRP) or intravitreal bevacizumab (IVB) injection. Diff: difference; CI: confidence interval

Variable	Time		Treatment		Diff	95% CI		*P*†
			PRP (Mean ± SD)	IVB (Mean ± SD)		Lower	Upper	
Whole disc								
	Baseline		45.5 ± 3.2	46.4 ± 3.4	-0.88	-2.75	0.99	0.357
	Month 1		44 ± 3.3	47.4 ± 2.8	-3.33	-5.09	-1.57	0.002
	Change 0–1		-1.54 ± 3.25	+ 0.99 ± 3.08	-2.45	-4.29	-0.61	0.009
	P-within‡		0.032	0.327				
	Month 3		44.2 ± 3.2	47.5 ± 3.3	-3.22	-1.27	-5.16	0.001
	Change 0–3		-1.32 ± 2.33	+ 1.15 ± 3.69	-2.63	-0.98	-4.28	0.002
	P-within‡		0.004	0.352				
	Change 1–3		-0.19 ± 2.41	+ 0.16 ± 2.89	-0.48	-1.85	0.9	0.497
	P-within‡		0.999	0.99				
Inside disc								
	Baseline		47.7 ± 6.1	43.1 ± 6.3	4.44	0.81	8.08	0.017
	Month 1		44.7 ± 5.6	45 ± 5.7	-0.5	-3.26	4.23	0.795
	Change 0–1		-2.99 ± 6.33	+ 1.85 ± 6.89	-4.58	-8.41	-0.75	0.019
	P-within‡		0.032	0.485				
	Month 3		44.7 ± 5.2	44 ± 7.7	1.02	-3.2	5.23	0.636
	Change 0–3		-3.68 ± 5.47	+ 0.89 ± 8.11	-4.68	-8.46	-0.91	0.015
	P-within‡		0.001	0.935				
	Change 1–3		-1.17 ± 4.59	-0.95 ± 6.68	-1.24	-3.21	0.72	0.215
	P-within‡		0.871	0.865				
Peripapillary vascular density								
	Baseline		47.7 ± 3.6	49.3 ± 4.4	-1.67	-0.06	3.95	0.153
	Month 1		45.8 ± 3.9	50.3 ± 3.1	-4.33	-6.38	-2.29	0.001
	Change 0–1		-1.85 ± 3.56	+ 0.95 ± 3.86	-2.95	-4.87	-1.06	0.002
	P-within‡		0.016	0.552				
	Month 3		45.7 ± 3.8	50.2 ± 3.9	-4.26	-6.63	-1.88	0.001
	Change 0–3		-1.83 ± 3.55	+ 0.9 ± 3.74	-2.74	-4.78	-0.69	0.009
	P-within‡		0.011	0.58				
	Change 1–3		-0.39 ± 2.93	+ 0.06 ± 3.05	-0.42	-1.97	1.14	0.599
	P-within‡		0.99	0.99				
RNFL thickness								
	Baseline		104.6 ± 17.5	112 ± 19.8	-5.15	-18.07	7.77	0.434
	Month 1		114.2 ± 15.6	109.3 ± 19.7	5.84	-6.39	18.06	0.349
	Change 0–1		+ 9.52 ± 7.32	-2.71 ± 5.46	-11.65	-7.5	-15.79	0.001
	P-within‡		0.002	0.58				
	Month 3		110.7 ± 16.3	107.4 ± 18	5.66	-6.15	17.47	0.348
	Change 0–3		+ 5.05 ± 7.07	-4.62 ± 5.55	+ 9.22	-4.93	-13.51	0.001
	P-within‡		0.001	0.001				
	Change 1–3		-4.63 ± 7.37	-1.9 ± 5.16	-2.81	-6.48	0.86	0.134
	P-within‡		0.009	0.001				

P-within‡: The difference between time points and baseline (change values) in each treatment group. P†: The difference between the IVB group versus the PRP group

In the PRP group, funduscopy indicated apparent regression of PDR in all eyes after 3 months, and no additional PRP was administered. By the third month, four eyes had a CMT exceeding 310 μm (none at the first month). Consequently, no additional IVB injections were given to the PRP group during the 3-month follow-up period; injections were administered only after the study had concluded.

### Vessel density and retinal nerve fiber layer (RNFL) thickness

**Fig. 2:**
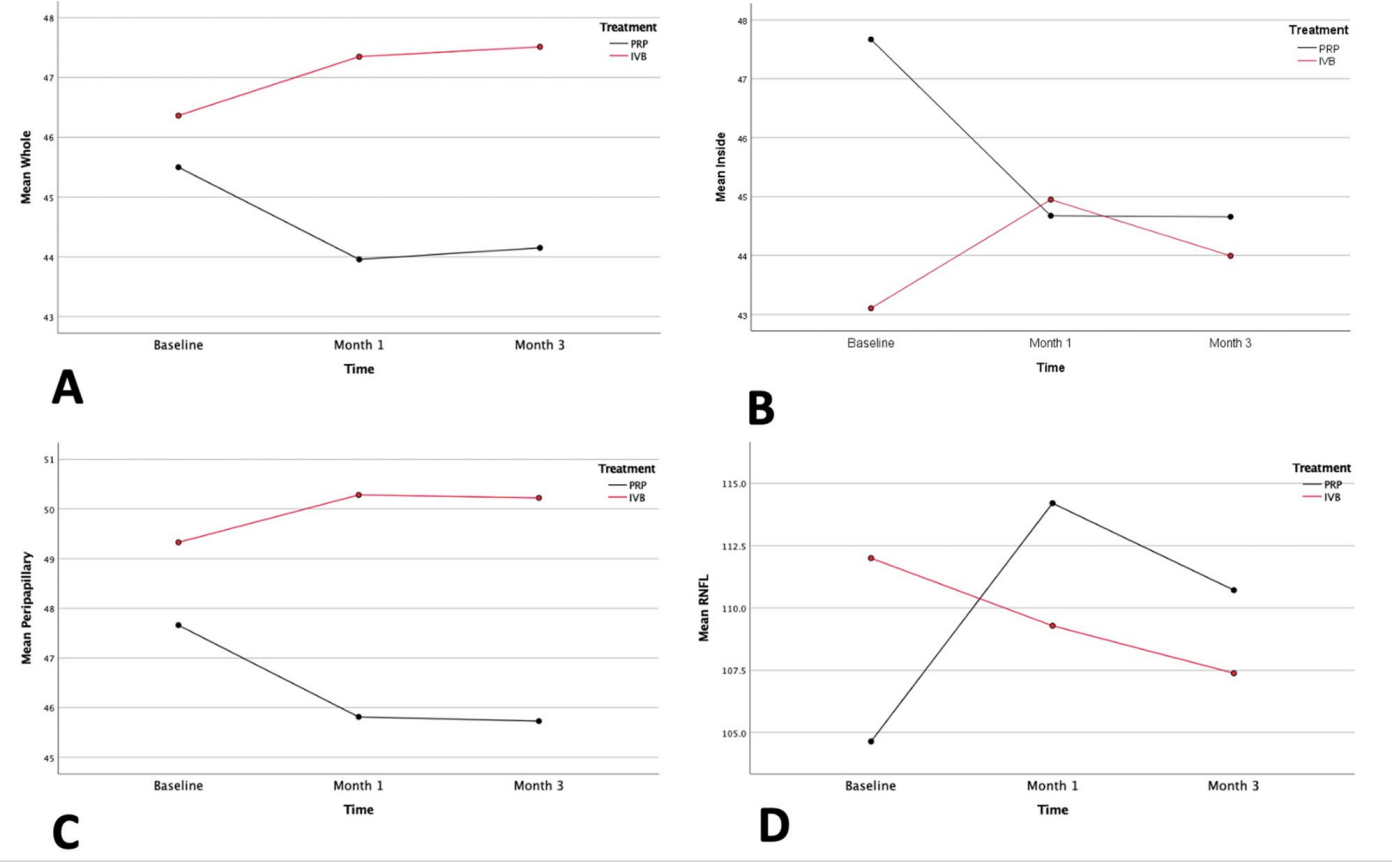
Diagrams of vascular density changes over time in the different regions in the two groups of the study. The red curve refers to intravitreal bevacizumab (IVB) treatment and the black curve refers to pan-retinal photocoagulation (PRP). All diagrams show that IVB treatment leads to an increase in vascular density and PRP leads to a decrease in vascular density. **A**: The whole vascular density changes. **B**: The inside vascular changes. **C**: The peripapillary region changes. **D**: The retinal nerve fiber layer (RNFL) thickness changes, showing that IVB causes a decrease in RNFL, and PRP initially increases it and decreases it between the first and third month

The changes in vascular density in different regions as well as RNFL thickness are shown in Table 2. Also, the comparison of vascular density change between two groups at different time points is shown in Fig. 2.

At baseline, there was no statistically significant difference in whole image capillary vascular density between the two treatment groups (45.5 ± 3.2 in PRP and 46.4 ± 3.4 in IVB; *p* = 0.36).

During follow-up, whole image capillary vascular density decreased in the PRP group at month 1 (44 ± 3.3) and month 3 (44.2 ± 3.2). This reduction was statistically significant in comparison with baseline values at both time points. (*p* = 0.032 and *p* = 0.004, respectively) The whole image capillary vascular density in the IVB group did not change significantly (month 1: 47.4 ± 3.3, *p* = 0.32; and month 3: 47.50 ± 3.3, *p* = 0.35). Whole image capillary vascular density changes were significantly different between treatment groups at month 1 (*p* = 0.009), and month 3 (*p* = 0.002).

The findings on the inside disc vascular density changes were very similar to the whole disc capillary vascular density. While there was no significant change in the IVB group (43.1 ± 6.3, 45.0 ± 5.7, and 44.0 ± 7.7 at baseline, month 1, and month 3, respectively), inside disc vascular density was decreased significantly in the PRP group during the first month and remained stable at month 3 (47.7 ± 6.1, 44.7 ± 5.6 (*p* = 0.032), and 44.7 ± 5.2(*p* = 0.001) at baseline, month 1, and month 3, respectively). The inside disc vascular density changes were significantly different between treatment groups at month 1 (*p* = 0.019), and month 3 (*p* = 0.015).

Prepapillary vascular density decreased significantly following PRP (baseline: 47.7 ± 3.6, month 1: 45.8 ± 3.9, and month 3: 45.7 ± 3.8, *P* = 0.016 and *p* = 0.011, respectively). In contrast, the Prepapillary vascular density increased slightly in the IVB group after 1 month (baseline: 49.3 ± 4.4, month 1: 50.3 ± 3.1, and month 3: 50.2 ± 3.9, *P* > 0.05 for all). A comparison of two treatment arms based on mean peripapillary vascular density change revealed a significant difference at month 1 and month 3 (*p* = 0.002 and *p* = 0.009, respectively).

At baseline, there was no statistically significant difference in RNFL thickness between the two treatment groups (104.6 ± 17.5 microns in PRP and 112 ± 19.8 microns in IVB group; *p* = 0.43).

Peripapillary RNFL thickness increased in the PRP group at month 1 (114.2 ± 15.6, *p* = 0.002) and then decreased at month 3 (110.7 ± 16.3 *p* = 0.001). During the 3-month follow-up, the peripapillary RNFL thickness decreased significantly in the IVB group (month 1: 109.3 ± 19.7, *p* = 0.58; and month 3: 107.4 ± 18, *p* = 0.001). Peripapillary RNFL thickness changes were significantly different between treatment groups at month 1 and month 3 (*p* = 0.001 for both).

The RNFL changes during the study did not significantly correlate with peripapillary VD changes in each group (*p* = 0.231 and *p* = 372, for PRP and IVB groups, respectively).

### Best corrected visual acuity (BCVA)

There was no significant difference between PRP and IVB groups based on BCVA, neither at the beginning of the study nor the end of the third month, (baseline BCVA: 0.52 ± 0.24 LogMAR in PRP group vs. 0.48 ± 0.25 LogMAR in IVB group, *p* = 0.52; month 3 BCVA: 0.54 ± 0.29 LogMAR in PRP group vs. 0.41 ± 0.20 LogMAR in IVB group, *p* = 0.25). In the PRP group, BCVA decreased slightly, but in the IVB group, there was a slight improvement in BCVA. However, the mean change was not statistically significant between the treatment groups. (0.01 ± 0.21 vs. 0.09 ± 0.17, *p* = 0.12) (Table 3).

**Table 3:** Best corrected visual acuity (BCVA) 1 month, and 3 months after pan-retinal photocoagulation (PRP) or intravitreal bevacizumab (IVB) injection. Diff: difference; CI: confidence interval

	Treatment					
	PRP (Mean ± SD)	IVB (Mean ± SD)	Diff	95% CI		*P*†
				Lower	Upper	
Baseline (LogMAR)	0.52 ± 0.24	0.48 ± 0.25	0.04	-0.10	0.18	0.524
Month 3 (LogMAR)	0.54 ± 0.29	0.41 ± 0.20	0.13	-0.02	0.28	0.253
BCVA Change (LogMAR)	0.01 ± 0.21	0.09 ± 0.17	-0.09	-0.21	0.02	0.126

P†: The difference between the IVB group versus the PRP group

### Central macular thickness (CMT)

Initial CMT measurements were comparable between the IVB and PRP groups (IVB: 255.13 ± 33.16 μm, PRP: 252.92 ± 24.36 μm; *P* = 0.8). Nevertheless, the PRP group exhibited a substantial increase in CMT at both one and three months (277.91 ± 43.25 μm, *P* < 0.01; 289.57 ± 60.76 μm, *P* = 0.03). Conversely, the IVB group did not show appreciable CMT changes over time (month 1: 258.48 ± 35.41 μm, *P* = 0.7; month 3: 266.18 ± 29.34 μm, *P* = 0.99). As a result, the CMT changes diverged significantly between the two groups at both time points (*P* = 0.01, *P* = 0.03).(Table 4).

**Table 4:** Central macular thickness(CMT) one month and three month after panretinal photocoagulation(PRP) or intravitreal bevacizumab(IVB) injection. Diff: difference; CI: confidence interval

Variable	Time		Treatment		Diff	95%CI		*P*†
			IVB(Mean ± SD)	PRP(Mean ± SD)		Lower	Upper	
CMT (µm)	Baseline	Value	255.13 ± 33.16	252.92 ± 24.36	2.02	–14.33	18.37	0.80
	Month 1	Value Change P-within ‡	258.48 ± 35.41 3.35 ± 17.01 0.70	277.91 ± 43.25 23.57 ± 35.21 0.00	–16.68 –18.53	–38.99 –33.49	5.62 –3.57	0.14 0.01
	Month 3	Value Change P-within ‡	266.18 ± 29.34 1.55 ± 18.43 0.99	289.57 ± 60.76 34.1 ± 58.37 0.03	–22.00 –30.64	–54.67 –59.51	10.66 –1.77	0.18 0.03

P-within ‡, The difference between time points and baseline(change values) in each treatment group; P†, the difference between IVB group versus PRP group

## Discussion

As previous studies have shown, retinal vascular density decreases with the progression of diabetic retinopathy (DR) [30, 31]. On the other hand, PRP as a treatment for PDR may lead to decreasing VD which is reported in several studies [19, 32, 33]. Nowadays, the potential of IVB treatment as an alternative treatment in severe conditions of DR like proliferative diabetic retinopathy (PDR) is being evaluated in several studies [2, 34, 35]. The IVB effects on increasing or decreasing VD of the optic disc are controversial among the studies, and there are sparse studies to compare the effect of these two treatment methods on optic disc microvasculature [14, 33, 36–38]. This study uses OCT-A to compare vascular density (VD) changes in the normal optic disc head, without neovascular tissue, along with RNFL alterations after two different interventions (IVB and PRP) at one and three months.

In the assessment of the VD changes by OCT-A in different regions of the optic nerve head between two interventions (IVB and PRP), we found that in the early period (first month after the interventions) the VD was changed significantly different among the two groups. Also, these differences remain constant at the end of the third month.

In this study, all the whole disc, inside disc, and peripapillary vascular densities were significantly decreased 1 month after PRP and remained stable during 3 months follow-up. On the other hand, there was an insignificant and slight increase in the eyes receiving IVB.

In a comparable study, Helmy and colleagues discovered that changes in mean (SD) RPC density from baseline to 1-, 2-, and 3-month follow-ups differed substantially between the PRP and intravitreal ranibizumab injection groups (all *P* < 0.05). The PRP group had a significantly larger mean (SD) RPC density changes from baseline to the 3-month follow-up than the ranibizumab group (− 2.16% [3.55%] versus 1.45% [2.59%]; *P* = 0.001). RPC density differed considerably between the two groups. Similar to the current investigation, over a three-month period, they determined that repeated assessments of radial peripapillary capillary (RPC) density show decreased RPC density in the PRP group and increased RPC density in the ranibizumab group [38]. In contrast to our study, treatment-naive eyes with PDR and NVD had been included in their study. Regression of NVD after these treatments may interfere with optic disc and peripapillary vascular density measurements.

Along with our study, Amanat et al. mentioned a significant reduction of optic nerve head VD, 1 month after PRP using OCTA in patients with PDR [19]. In another recently published study by Zhao et al. it was shown that after PRP treatment, there was no significant change in peripapillary VD after 12 months. Although, they did not report the early vascular density changes after PRP.^33^ Also, they showed that after intravitreal Conbercept injection at the end of the 12th month, there was a very small and insignificant increase in peripapillary VD and inside disk VD [33]. The difference between the two studies may be attributable to the use of distinct anti-VEGF types, intravitreal injection quantities, or follow-up durations.

Although the mechanism of action of PRP remains unclear, The main concept is that PRP acts by destroying the peripheral retinal pigment epithelium (RPE) and adjacent photoreceptors to diminish retinal oxygen consumption and increase oxygen diffusion into the vitreous cavity to reduce the hypoxia of the posterior retina [39]. The improved oxygenation of the retina leads to constriction of the posterior retinal arterioles [40]. Along with this theory, Wilson et al. found that the mean retinal arteriolar diameter decreased by nearly 10% after PRP treatment in PDR eyes [41]. Afterward, Mendrinos et al. illustrated that the retinal arteriolar diameter decreased by 13.8% following PRP in NPDR and PDR eyes, using a retinal vessel analyzer on serial fundus photos. Theoretically, the vasoconstriction should result in a decrease in VD measurement [40]. There is no evidence suggesting that this PRP-induced vasoconstriction is permanent. Indeed, it may be due to retinal autoregulatory mechanisms. Zhao and his colleagues also could not show any significant peripapillary VD reduction after 1 year [33]. Some earlier studies suggested a possible mild increase in cup-to-disc ratio and optic disc pallor in eyes receiving PRP [42]. Although vasoconstriction of inside disc microvessels may be responsible for this appearance, some attributed these findings to thinning of the RNFL [8]. It is worth noting that diabetic subjects with and without PRP were more likely to have optic disc graded as abnormal compared with control subjects [43].

VEGF inhibition has been reported to induce retinal arteriolar constriction and the vasoconstriction was a concern that might increase hypoxic damage to retina capillaries and lead to further ischemia [44, 45]. However, our results revealed that the whole disc along with the inside disc and peripapillary vascular densities did not change significantly after the IVB treatment. Therefore, we conclude that there is no reason to be concerned that anti-VEGF therapy may exacerbate peripapillary ischemia in eyes with diabetic retinopathy. Similar to our findings, Zhao et al. revealed that macular and papillary VD did not change significantly after the intravitreal Conbercept injections [33]. 

Another parameter that may be affected by the different treatment regimens is RNFL thickness. The findings of the current investigation indicate that PRP results in a significant elevation of RNFL thickness within the initial month of intervention. However, as time progresses, the thickness of RNFL experiences a slight decline until the third month. Conversely, the administration of IVB results in a reduction in retinal nerve fiber layer (RNFL) thickness during the initial three-month period. The observed variations were notably distinct across these cohorts, both during the initial and subsequent months. Although the changes were significantly different between the groups during these 3 months but the thickness of RNFL was not significantly different between the treatment groups at month 3. Over a period of ten months, Roohipour et al. evaluated the effects of two treatment regimens (PRP and PRP + IVB) on 64 eyes of PDR patients. As a consequence, they reported that RNFL initially increased marginally following PRP. However, there was no statistically significant difference between the PRP group and the PRP + IVB group, and after ten months, RNFL thickness in both groups was comparable to that at baseline [46]. The presence of diabetes itself can cause neurodegenerative changes in the retina. Studies using diabetic rats show a loss of retinal ganglion cells and thinning of the retinal inner and outer plexiform nuclear layers and a loss of retinal axons [43, 47]. In vivo, photographic studies show loss of the nerve fiber layer, which correlates with the severity of diabetic retinopathy [48]. Lim et al. showed that PRP may thin the RNFL beyond the effects of diabetes alone, and optic nerves in eyes treated with PRP are more likely to be graded as abnormal, but they couldn’t rule out the worsening of retinal ischemia in eyes with proliferative diabetic retinopathy after PRP [8]. Along with our study, Entezari et al. showed that RNFL thickness may decrease temporarily after 3 months following IVB injections in patients with wet-type age-related macular degeneration (ARMD) [49]. Martinez-de-la-Casa et al. [50] and Ahn et al. [51] also reported a significant reduction in global RNFL thickness in eyes with multiple anti-VEGF injections. Some previous investigations showed that IOP fluctuation after multiple injections does not adversely affect the RNFL thickness [52, 53]. In our study, although the mean RNFL thickness showed a significant decrease, it was within 5 μm, which is not clinically significant. It is not considered that the observed reduction in the mean RNFL thickness of the treated eyes was an adverse effect of the anti-VEGF injection. This reduction may instead be related to the anatomical changes of macular area after the three injections. [51] It has been shown that the peripapillary RNFL thickness was increased in patients with diabetic macular edema (DME) [54]. The transient increase in RNFL thickness following PRP treatment may be attributed to factors such as impaired axonal flow and intraretinal inflammation [55, 56].The RNFL changes during the study did not significantly correlate with peripapillary VD changes in each group.

Our study assess the impact of PRP and IVB on visual function. Over a three-month follow-up, we found no significant difference in BCVA between the two groups. While the PRP group experienced a slight decrease in BCVA by the third month, the IVB group showed a minor improvement. However, this difference was not statistically significant (*p* = 0.12). These findings suggest that although PRP and IVB can alter structural parameters like vascular density and RNFL thickness, these changes may not directly translate to short-term visual function. Future long-term studies are needed to determine whether these structural changes may influence vision over an extended period.

### Limitations of the study

There were limitations to this investigation. First, the retrospective design may introduce inherent biases. The limited follow-up period is another significant drawback, and additional follow-up is required to evaluate long-term changes and outcomes. The small sample size is another limitation that will need to be investigated with larger groups in the future. The lack of functional correlation limits insights into how these structural changes impact visual function. Additionally, the use of a bevacizumab biosimilar rather than the reference drug or other anti-VEGF agents may restrict comparisons with other treatments in this class. Diabetic macular edema (DME) may act as a potential confounding factor influencing RNFL thickness. Furthermore, the lack of baseline data on metabolic factors beyond HbA1C—such as blood pressure, lipid profile, and others—among our patients may have influenced their response to treatments. Finally, this study focused on eyes without high-risk proliferative retinopathy characteristics, and thus, the findings cannot be applied to eyes with high-risk PDR.As mentioned in the limitations, While our study provides valuable insights into the early effects of PRP and IVB on optic disc microcirculation and RNFL thickness, the importance of functional implications of the observed structural changes should be considered and further research with longer follow-up is needed to explore the potential impact of these changes on visual function.

## Conclusion

PRP significantly reduced vascular density in the optic disc and peripapillary regions at both one and three months. While RNFL thickness showed an initial increase at one month but declined by the third month. In contrast, IVB resulted in a slight increase in optic disc vascular density and a significant reduction in RNFL thickness. Notably, the changes in RNFL thickness did not correlate with peripapillary vascular density changes in either group. These findings underline the distinct effects of PRP and IVB on optic nerve microvasculature, emphasizing the need for further investigation into the long-term effects of these treatments.

## Data Availability

The datasets generated during and/or analyzed during the current study are available from the corresponding author on reasonable request.

## Abbreviations

DR: Diabetic retinopathy
NPDR: Non-proliferative diabetic retinopathy
PDR: Proliferative diabetic retinopathy
PRP: Pan-retinal photocoagulation
VEGF: Vascular endothelial growth factor
IVB: Intravitreal injections of bevacizumab
DME: Diabetic macular edema
OCT: Optical coherence tomography
OCTA: Optical coherence tomography angiography
VD: Vessel density
RNFL: Retinal nerve fiber layer
RPC: Radial peripapillary capillary
LogMAR: Logarithms of the minimum angle of resolution
BCVA: Best corrected visual acuity
CMT: Central macular thickness
NVD: Neovascularization of the optic disc
FA: Fluorescein angiography
SSADA: Split-spectrum amplitude-decorrelation angiography
PAR: Projection artifact removal
GEE: Generalized estimating equation
ILM: Internal limiting membrane
ARMD: Age-related macular degeneration
